# Effect of a brief cognitive behavioral program on depressive symptoms among newly licensed registered nurses: An observational study

**DOI:** 10.1371/journal.pone.0240466

**Published:** 2020-10-12

**Authors:** Kosei Esaki, Masashi Ikeda, Tomo Okochi, Satoru Taniguchi, Kohei Ninomiya, Ayu Shimasaki, Yasuyo Otsuka, Yoshiko Oda, Takaya Sakusabe, Keiko Mano, Takeo Saito, Nakao Iwata

**Affiliations:** 1 Department of Psychiatry, Fujita Health University School of Medicine, Toyoake, Aichi, Japan; 2 Division of Nursing, Fujita Health University Hospital, Toyoake, Aichi, Japan; 3 Medical Engineering, Fujita Health University School of Medical Sciences, Toyoake, Aichi, Japan; University of Toronto, CANADA

## Abstract

Depressive symptoms are a serious problem in workplaces. Hospital staff members, such as newly licensed registered nurses (NLRNs), are at particularly increased risk of these symptoms owing to their limited experience. Previous studies have shown that a brief program-based cognitive behavioral therapy program (CBP) can offer effective treatment. Here, we conducted a longitudinal observational study of 683 NLRNs (CBP group, n = 522; no-CBP group, n = 181) over a period of 1 year (six times surveys were done during this period). Outcomes were assessed on the basis of surveys that covered the Beck Depression Inventory-I (BDI). The independent variables were CBP attendance (CBP was conducted 3 months after starting work), personality traits, personal stressful life events, workplace adversity, and pre-CBP change in BDI in the 3 months before CBP (ΔBDI_pre-CBP_). All factors were included in Cox proportional hazards models with time-dependent covariates for depressive symptoms (BDI ≥10), and we reported hazard ratios (HRs). Based on this analysis, we detected that CBP was significantly associated with benefit for depressive symptoms in all NLRNs (*P*_uncorrected_ = 0.0137, HR = 0.902). To identify who benefitted most from CBP, we conducted a subgroup analysis based on the change in BDI before CBP (ΔBDI_pre-CBP_). The strongest association was when BDI scores were low after starting work and increased before CBP (*P*_uncorrected_ = 0.00627, HR = 0.616). These results are consistent with previous findings, and indicate that CBP may benefit the mental health of NLRNs. Furthermore, selective prevention based on the pattern of BDI change over time may be important in identifying who should be offered CBP first. Although CBP is generally effective for all nurses, such a selective approach may be most appropriate where cost-effectiveness is a prominent concern.

## Introduction

A deterioration in subjective well-being, or more specifically the onset of depressive symptoms, is a worldwide challenge in many workplaces. Such conditions are not only very common [1] but also have significant economic, productivity, and quality of life implications, especially if workers develop depression [2].

Depressive symptoms are relevant to all workers in all occupations, but they are a particular problem among hospital staff [3–5]. Due to the expectations placed on nurses, who must remain highly motivated in the face of significant responsibility, professional conflict, and the strains of night shifts, among other factors, this group is at particularly increased risk [6]. Moreover, as newly licensed registered nurses (NLRNs) tend to be young and have less experience, they may lack the skills and confidence to cope with workplace stress. In turn, this may place them at higher risk of depressive symptoms or worse well-being [7]. Improved training in coping skills could help to maintain well-being in this group, protecting them against psychologic distress, depression, and burnout.

Cognitive behavioral therapy (CBT) is a sophisticated psychological therapy with long-term efficacy, and it has become one of the most common and useful treatment options for many psychiatric disorders, including depression and anxiety. In the workplace, CBT has been modified to fit the needs of workers by using fewer sessions and focusing on the key facts for preventing psychological distress or maintaining well-being, as exemplified by cognitive behavioral programs (CBPs) [8]. To date, several internet-based and face-to-face intervention studies have been conducted, together with a couple of meta-analyses, and these suggest that CBT or CBP can prevent depressive symptoms and/or increase well-being and self-esteem [9–12]. A couple of studies have also targeted nurses and concluded that CBT might be effective, although the evidence was of low quality [13, 14].

In this study, we aimed to confirm whether CBT/CBP was an effective intervention for nurses and to identify those nurses in whom CBP has the greatest effect. Therefore, we analyzed longitudinal data for NLRNs in Japan based on reported depressive symptoms. Although this was an observational study, we considered that it was possible to speculate on the effect of CBP by comparing the psychological distress scores on the Beck Depression Inventory-I (BDI-I) between subjects who did or did not attend CBP sessions.

## Materials and methods

### Subjects and data collection

Data were collected in six phases as part of the Depression Protection Program in Fujita [15]. We enrolled 910 NLRNs employed by Fujita Health University Hospital and starting work between 2012 and 2017 who agreed to participate. A subset of these subjects had been used in a previous analysis [15]. Inadequate questionnaire responses were provided by 227 subjects (i.e., did not respond to the “*baseline−1*,” “*baseline 0*,” or “all” for the follow-up surveys), so they were excluded. The ethics committee of Fujita Health University approved this study. After a complete description of the study was provided to the subjects, all gave their written informed consent.

In total, 683 subjects were eligible for inclusion, comprising 618 women and 65 men with a mean age of 22.2 ± 1.8 years at enrollment. These were divided into six phases by the year they started work: phase I, from April 2012 (n = 77); phase II, from April 2013 (n = 84); phase III, from April 2014 (n = 139); phase IV, from April 2015 (n = 109); phase V, from April 2016 (n = 125); and phase VI, from April 2017 (n = 149).

Subjects were evaluated every 3 months after the baseline assessment in April (i.e., July, October, January, and April the following year). As such, a person could be surveyed a maximum of six times: “*baseline−1*” (April in their first year, just after starting work), “*baseline 0*” (July in their first year, just before CBP), “Survey 1” (September in their first year: first time after CBP),”Survey 2” (October in their first year),”Survey 3” (January in their first year),”Survey 4” (April in their second year) (S1 Fig). Surveys were numbered with reference to when CBT was performed. Nurses were enrolled at *baseline−1*, when they were not working in clinical settings. The *baseline 0* survey was conducted just before CBT for subjects in phases III to VI, as described below.

Each subject was evaluated for the following: depressive symptoms, using the Beck Depression Inventory-I (BDI) [16]; stressful life events (SLEs) during the previous 6 months, based on the List of Threatening Experiences (LTE) 12-item questionnaire [17]; and workplace-based adversity, using our own questionnaire that covered exposure to intimidating doctors or patients, receiving abusive language, violence or sexual harassment, and involvement in medical incidents or accidents. Personality traits were assessed at enrollment using the NEO Five-Factor Inventory (NEO-FFI) [18].

### Cognitive behavioral program

In the Depression Protection Program in Fujita [15], CBP was introduced from 2014 as a part of the training and educational session provided by the Department of Nursing in Fujita Health University Hospital. Therefore, all subjects in phases III–VI received CBP (*N* = 522), whereas those in phases I and II did not receive CBP (*N* = 161). In S1 Table, the descriptive statistics of demographic characteristics for these subjects are shown.

The CBP was conducted in face-to-face group session (four times) by two trained psychologists. Sessions were limited to 1 hour each, once a week, with a maximum of ten subjects. The following content was covered: (1) understanding motives for change and stress/stressor, (2) understanding the ABC model and cognitive therapy, (3) training in cognitive restructuring to change their relationship with negative automatic thoughts, and (4) training to improve coping skills and to identify areas for possible behavioral change. The CBP was first delivered 3 months after starting work in the first year (August/September; S1 Fig).

### Statistical analysis

Demographic data were analyzed using *t*-test for continuous variables and chi-square test for categorical data using SPSS version 27 (IBM, New York, NY, USA).

The main analysis that was used to assess the effect of CBP was based on the three Cox proportional hazards models with time-dependent covariates, including person surveys as the unit of the analysis (rms package in R version 3.5.3: www.r-project.org). This analysis is expected to adjust numerous patterns of changes associated with the CBP group. Each model was based on BDI as the outcome variable, assigning 0 to scores <10 and 1 to scores ≥10 (indicating mild depressive symptoms [19]). The following were included as independent variables: prior CBP attendance, personality (i.e., neuroticism and openness scores), SLE count, workplace adversity count, sex, BDI score at baseline *0*, and pre-CBP change in BDI (ΔBDI_pre-CBP_) from *baseline–1* to *baseline 0*. This included their interactions: neuroticism × SLE count, neuroticism × workplace adversity, and CBP × ΔBDI_pre-CBP_. Personality scores, sex, and ΔBDI_pre_-CBP were treated as time-fixed covariates, and the other parameters as time-dependent covariates.

Before implementing the models, we checked for correlations among the NEO-FFI personality traits (i.e., neuroticism, extraversion, openness, agreeableness, and conscientiousness) using SPSS version 27 (IBM) to minimize collinearity (S2 Table). We also checked the ΔBDI_pre_-_CBP_ to assess the effect of work on the BDI (i.e., *baseline–1* to *baseline 0;* S2 Fig) because the response to the stressor might reflect the ΔBDI_pre-CBP_ and constitute a “personality” trait. Thus, we classified ΔBDI_pre-CBP_ groups as follows: (1) a low-to-high group whose BDI changed from <10 at *baseline−1* to ≥10 at *baseline 0* (no CBP = 110; CBP = 383), (2) a high-to-high group whose BDI remained ≥10 between *baseline−1* and *baseline 0* (no CBP = 45, CBP = 121), (3) a low-to-low group whose BDI remained <10 between *baseline−1* and *baseline 0* (no CBP = 6, CBP = 16), and (4) a high-to-low group whose BDI changed from ≥10 at *baseline−1* to <10 at *baseline 0* (no CBP = 0, CBP = 2; this group was therefore too small for analysis).

The detailed setting was as follows. As reported in previous studies [20, 21], neuroticism, a strong predictor, was modeled using a restricted cubic spline with five knots to allow for a potential nonlinear association, using the rcs function in the rms package in R program. We then scored SLE and workplace adversity as follows: 0, no event; 1, one event; and 2, at least two events. Finally, the ΔBDI_pre-CBP_ classifications were scored as follows: 1, low-to-low group and high-to-low group; 2, high-to-high group; and 3 low-to-high group. This order was used because we assumed that it would reflect an increase in benefit by CBP. The low-to-low and high-to-low groups were merged because of the small number in the latter and implied lack of efficacy of the CBP.

The type I error rate was set at 0.05. In this paper, uncorrected *P*-values were presented; although, we recognize that our results have multiple testing issues.

## Results

In total, 1804 surveys were completed by 683 subjects and were available for the analysis; of these, 161 had not received CBP and 522 had received CBP.

First, we checked the trends in the BDI scores for each survey, as shown in S3 Fig. Interestingly, the shape of the histogram changed from *baseline−1* (first survey after starting work) to *baseline 0* (pre-CBP) (Wilcoxon signed test: Z = −12.254, P = 1.60 × 10^−34^). However, there were no dramatic changes from *baseline−0* to Survey 1 (Z = -0.127, P = 0.899), from Survey 1 to Survey 2 (Z = −2.306, P = 0.0211), from Survey 2 to Survey 3 (Z = −2.789, P = 0.00529), and from Survey 3 to Survey 4 (Z = −1.458, P = 0.145).

Second, we confirmed the correlation of the NEO-FFI personality traits evaluated at enrollment. This analysis showed that the only traits that had no significant correlation were neuroticism (reported to be a strong risk factor [20, 21]) and openness (S2 Table). These were selected as independent variables for the subsequent analysis.

Cox proportional hazards analyzes were performed to assess the association between BDI and the effect of CBP. Despite our observational study design, we used the subjects who did not receive CBP in their first year after starting work as a comparison group in the model (i.e., those in phases I and II; n = 161). There was a significant association between BDI (≥10 or <10) and CBP attendance (P = 0.0137), the neuroticism score (P = 1.61 ×10^−7^), workplace adversity count (P = 0.0246), BDI score at *baseline 0* (P = 6.96 ×10^−14^), ΔBDI_pre-CBP_ (P = 5.82 ×10^−6^), and the interaction with ΔBDI_pre-CBP_ (P = 0.00750) (Table 1). Finally, because we detected significant differences for the ΔBDI_pre-CBP_ and the interaction of CBP and ΔBDI_pre-CBP_, we performed further analysis to confirm which sub-group of the ΔBDI_pre-CBP_ contributed most to the significant association between depressive symptoms and CBP. There was only a significant association with CBP in the low-to-high ΔBDI_pre-CBP_ group, which was expected given these were most sensitive to stressors (P = 0.00627; Table 2)

**Table 1:** Cox proportional hazards model for predicting the development of depressive symptoms in all subjects.

Factor	P-value	Hazard Ratio (95%CI)
CBP	**0.0137**	0.902 (0.718–1.13)
Neuroticism	**1.61x10**^**-7**^	2.17 (1.42–3.33)
Openness	0.508	0.954 (0.828–1.10)
SLE count	0.122	1.25 (0.680–2.30)
workplace adversity count	**0.0246**	1.60 (1.02–2.52)
sex	0.830	0.964 (0.690–1.35)
BDI (“*baseline0*”)	**6.96x10**^**-14**^	1.78 (1.53–2.07)
ΔBDI_pre-CBP_	**5.82x10**^**-6**^	3.18 (1.93–5.26)
CBP x “ΔBDI_pre-CBP_	**0.00750**	-
Neuroticism x SLE count	0.0826	-
Neuroticism x workplace adversity count	0.493	-

The hazard ratios for the main effects were calculated in the model without interactions because hazard ratios in the interaction model could be influenced by an "interaction effect.” Bold numbers show P-values <0.05.

Abbreviations: BDI, Beck Depression Inventory score; CBP, attendance at a Cognitive Behavioural Program; SLE, stressful life event.

**Table 2:** Cox proportional hazards model for predicting the development of depressive symptoms for subjects by the pattern of change in BDI before CBP.

	Pattern of change in BDI before CBP (ΔBDI_pre-CBP_ sub-group)					
	“Low to High” subjects		“High to High” subjects		“Low to Low” subjects	
Factor	P-value	Hazard Ratio (95%CI)	P-value	Hazard Ratio (95%CI)	P-value	Hazard Ratio (95%CI)
CBP	**0.00627**	0.616 (0.432–0.863)	0.965	0.992 (0.709–1.39)	0.879	0.962 (0.582–1.59)
Neuroticism	0.204	1.37 (0.732–2.12)	0.322	1.78 (0.583–5.42)	0.328	2.53 (0.987–6.52)
Openness	0.723	0.960 (0.790–1.24)	0.982	0.998 (0.830–1.20)	0.318	0.856 (0.632–1.16)
SLE count	0.021	1.24 (0.659–2.57)	0.275	1.40 (0.565–3.47)	0.0556	2.45 (0.913–6.55)
workplace adversity count	0.0989	1.66 (0.916–3.09)	**0.0413**	1.85 (1.00–3.41)	**0.0494**	2.40 (1.27–4.53)
sex	0.314	0.781 (0.480–1.33)	0.826	1.06 (0.615–1.84)	0.377	1.33 (0.707–2.50)
BDI (“*baseline0*”)	0.0711	1.28 (0.872–2.22)	0.305	0.905 (0.749–1.09)	0.607	1.19 (0.607–2.35)
Neuroticism*SLE count	0.142	-	0.342	-	0.733	-
Neuroticism*workplace adversity count	0.694	-	0.136	-	0.256	-

The hazard ratios for the main effects were calculated in the model without interactions because hazard ratios in the interaction model could be influenced by an "interaction effect.” Bold numbers show P-values <0.05.

Abbreviations: BDI, Beck Depression Inventory score; CBP, attendance at a Cognitive Behavioural Program; SLE, stressful life event.

## Discussion

This longitudinal observational survey with follow-up every 3 months confirmed that CBP [13, 14], neuroticism [20, 21], and recent workplace adversity were associated with depressive symptoms. Subgroup analysis further revealed that subjects who developed psychological distress after starting work gained the greatest benefit from CBP.

The Cox proportional hazards analysis of the effect of CBP indicated that there was a significant association between CBP and mild depressive symptoms for all NLRNs. Therefore, we consider that CBP will be of use as a universal prevention or intervention course. Previous research in a similar target population (NLRNs), but using a randomized control design, also revealed that CBP was effective against depressive symptoms in their entire cohort [13]. Our results support this finding and strengthen the evidence in favor of using CBP for NLRNs.

As a more selective intervention, we also showed that a low-to-high ΔBDI_pre-CBP_ sub-group was most likely to benefit from CBP (i.e., those whose BDI score changed from below to above the 10-point cutoff). This result was somewhat expected because such subjects were probably more sensitive to workplace stressors due to lower levels of resilience. Therefore, this study provides an indicator for selecting the most appropriate target population. Based on our assumption, approximately 70% (383 out of 522 subjects in the CBP subjects) of the subjects corresponded to this group, enabling us to reduce the cost by identifying appropriate targets with high possibility of responding to CBP.

Besides the main findings of CBP effect, the trends in the BDI scores for each survey revealed interest implications: We clarified the time course of depressive symptoms based on the BDI score, with evidence showing that scores tended to deteriorate after starting the work (*baseline 0*), remained somewhat higher for half a year and then stabilized. This implied that interventions to prevent depressive symptoms, such as CBP, should be planned early after starting work [13], consistent with our approach.

On the basis of these findings, CBP at an appropriate time can be a recommended program for NLRNs and probably, for general workers who are new employees, to prevent depressive symptoms. Also, CBP may influence subjects' cognition temporarily and permanently; therefore, the subjects who attend CBP would acquire protective skill against depression for many years, especially in subjects who are sensitive to stresses.

Our study contains important limitations that must be considered when interpreting the results. First, as this was a longitudinal observational study, the no-CBP group was not regarded as the best control group. This is because we did not conduct a randomized control trial (RCT), and our CBP was carried out as a part of training and educational sessions. Although we recognize that the “no-CBP group” in our study has several disadvantages, it is important to mention that there were no obvious difference in the clinical background of CBP and no-CBP subjects and this supports our decision that no-CBP group can be considered as “historical controls” (S1 Table). Second, we did not correct the issue of multiple comparisons. It is important to mentioned that there is no gold standard for correction in such correlated variables, and therefore we present uncorrected P-values throughout the study. However, we are confident of our results as they are in line with previous studies [13, 14], providing an additional proof of replication of the CBP effect. Third, not all subjects responded to the surveys, which introduced loss to follow-up and potential bias. Fourth, the comparison sample (phases I and II) with no CBP was small. There is a need for further study in a larger sample size.

In conclusion, our data support the results of previous research in indicating that CBP is probably beneficial for NLRNs. We add to this knowledge base by showing that a change in the BDI over a 3 month period after first starting work (i.e., ΔBDI_pre-CBP_) can be used to identify the most suitable target population. Specifically, our data indicate that those who change from a low to a high BDI score (i.e., the low-to-high ΔBDI_pre-CBP_ sub-group) were most likely to benefit from CBP.

## Supporting information

S1 Fig Scheme of our study. (TIFF)

S2 Fig Pre-CBP change in BDI (ΔBDI_pre-CBP_) from *baseline–1* to *baseline 0*. (TIFF)

S3 Fig Trends in the BDI scores for each survey. (TIFF)

S1 Table The descriptive statistics of demographic characteristics in no-CBP and CBP groups. (DOCX)

S2 Table Correlation among subsets of NEOAC. (DOCX)
