# Supplementary figures and images for: Effect of a brief cognitive behavioral program on depressive symptoms among newly licensed registered nurses: An observational study
Source: PLoS One. 2020 Oct 12;15(10):e0240466. doi: 10.1371/journal.pone.0240466 (PMC7549829; doi:10.1371/journal.pone.0240466)

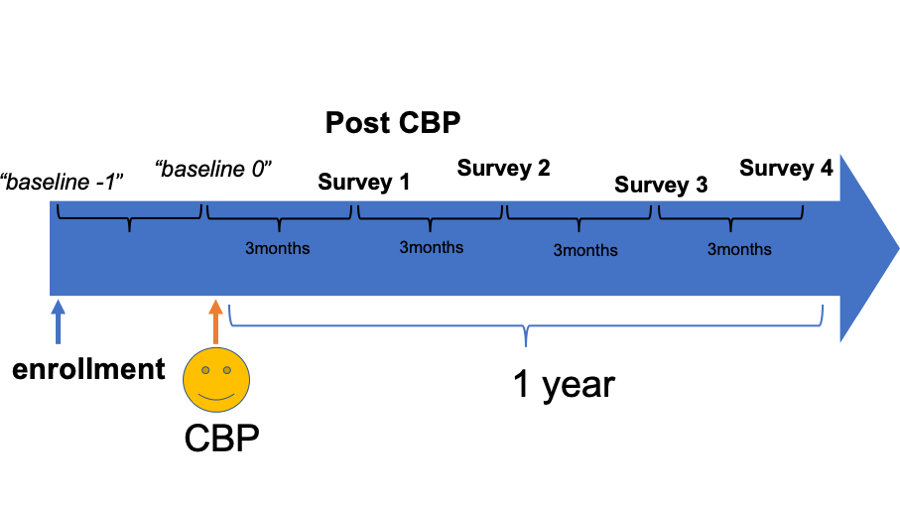

Supplement: S1 Fig — (TIFF) [file pone.0240466.s001.tiff]

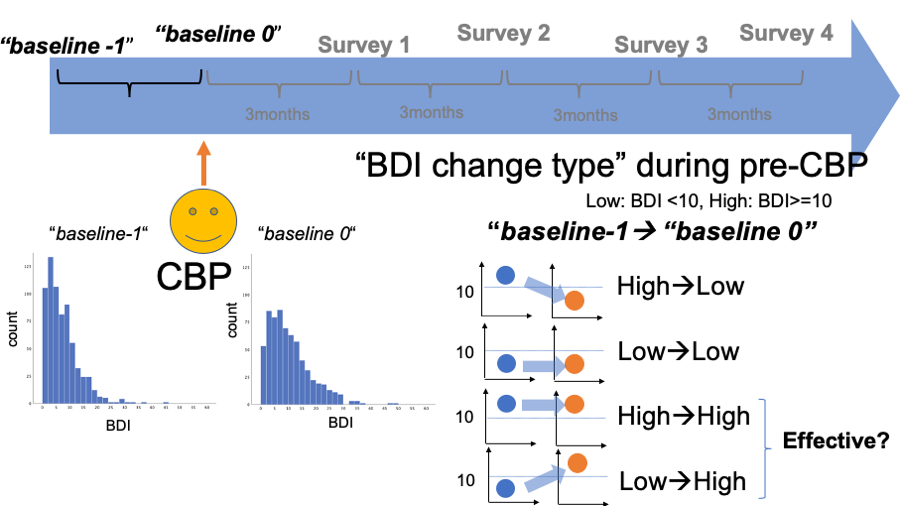

Supplement: S2 Fig — (TIFF) [file pone.0240466.s002.tiff]

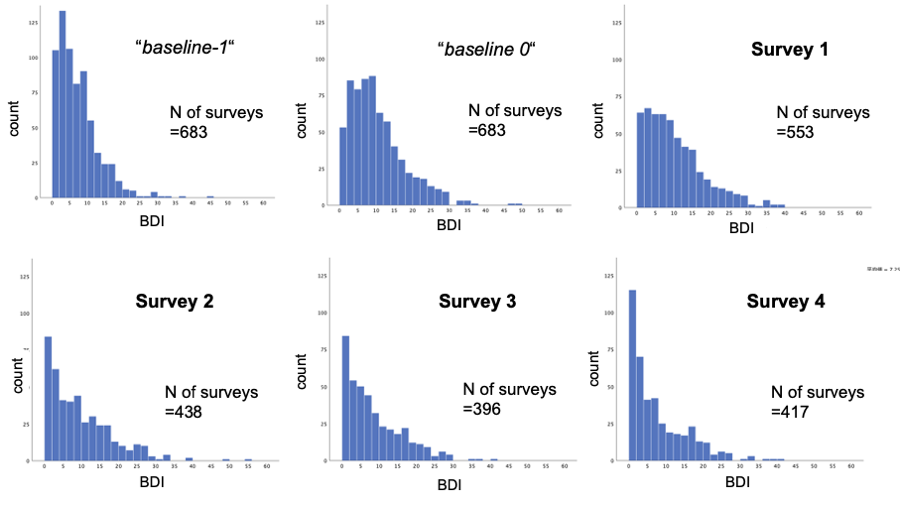

Supplement: S3 Fig — (TIFF) [file pone.0240466.s003.tiff]
