# Supplementary material for: Effect of a brief cognitive behavioral program on depressive symptoms among newly licensed registered nurses: An observational study
Source: PLoS One. 2020 Oct 12;15(10):e0240466. doi: 10.1371/journal.pone.0240466 (PMC7549829; doi:10.1371/journal.pone.0240466)
Supplement: S1 Table — (DOCX) [file pone.0240466.s004.docx]

|  | no-CBP group (mean ± SD) | CBP group (mean ± SD) | *t*-value | df | *P* |
| --- | --- | --- | --- | --- | --- |
| age | 22.34 ± 2.39 | 22.09 ± 1.62 | 1.262 | 207.216 | 0.208* |
| neuroticism | 56.94 ± 9.66 | 56.91 ± 10.03 | 0.036 | 681 | 0.971 |
| extraversion | 51.4 ± 9.93 | 50.95 ± 9.96 | 0.5 | 681 | 0.617 |
| openness | 51.52 ± 7.82 | 49.72 ± 8.51 | 2.393 | 681 | 0.017 |
| agreeableness | 50.65 ± 10.87 | 49.92 ± 9.78 | 0.806 | 681 | 0.42 |
| conscientiousness | 47.4 ± 9.92 | 47.17 ± 9.68 | 0.259 | 681 | 0.796 |
| BDI ("baseline-1") | 7.11 ± 6.77 | 6.61 ± 5.47 | 0.856 | 228.126 | 0.393* |
| BDI ("baseline0") | 9.91 ± 7.42 | 9.91 ± 7.44 | −0.002 | 681 | 0.999 |
|  |  |  | chi-squared | df | P |
| sex | 48/17 (female/male) | 474/144 (female/male) | 0.13094 | 1 | 0.7175 |

Table S1: The descriptive statistics of demographic characteristics in no-CBP and CBP groups

*This value is based on the assumption that the variances are not equal (significant Levene Test for equality of variances [*P* < 0.05]).

CBP: cognitive behavioral program
