# Supplementary material for: Effect of a brief cognitive behavioral program on depressive symptoms among newly licensed registered nurses: An observational study
Source: PLoS One. 2020 Oct 12;15(10):e0240466. doi: 10.1371/journal.pone.0240466 (PMC7549829; doi:10.1371/journal.pone.0240466)
Supplement: S2 Table — (DOCX) [file pone.0240466.s005.docx]

|  |  |  |  |  |  |  |
| --- | --- | --- | --- | --- | --- | --- |
|  |  |  |  | Pearson r |  |  |
|  |  | neuroticism | extraversion | openness | agreeableness | conscientiousness |
|  | neuroticism | 1 | -0.293 | 0.0222 | -0.304 | -0.180 |
|  | extraversion | P=5.9x10^-15^ | 1 | 0.176 | 0.333 | 0.226 |
| P value | openness | P=0.56 | P=3.7x10^-6^ | 1 | 0.115 | 0.0152 |
|  | agreeableness | P=4.5x10^-16^ | P<2.2x10^-10^ | P=0.0260 | 1 | 0.170 |
|  | conscientiousness | P=2.1x10^-6^ | P=2.5x10^-9^ | P=0.691 | P=8.4x10^-6^ | 1 |
|  |  |  |  |  |  |  |

S2 Table: Correlation among subsets of NEOAC
